# Efficacy of Rectal Misoprostol for Prevention of Postpartum Hemorrhage

**Published:** 2013

**Authors:** Masoumeh Mirteimouri, Fatemeh Tara, Batool Teimouri, Nahid Sakhavar, Afsaneh Vaezi

**Affiliations:** a *Department of Obstetrics and Gynecology, Omolbanin Hospital, School of Medicine, Mashhad University of Medical Sciences, Mashhad, Iran.*; b *Department of Obstetrics and Gynecology, Zahedan University of Medical Sciences, Zahedan, Iran.*; c *Gynecologist, Zahedan, Iran. *

**Keywords:** Misoprostol, Oxytocin, Postpartum hemorrhage, drug

## Abstract

Postpartum hemorrhage is an important cause of maternal morbidity and mortality after delivery. Active management of postpartum hemorrhage by an uterotonic drug decreases the rate of postpartum hemorrhage. The aim of this study is to evaluate the efficacy of rectal misoprostol for prevention of postpartum hemorrhage. This double blind randomized clinical trial was performed on full term pregnant women candidate for vaginal delivery, referred to Zahedan Imam Ali Hospital during 2008-2009. They were randomly divided into two groups of rectal misoprostol and oxytocin. The women in misoprostol group received 400 μg rectal misoprostol after delivery and the women in oxytocin group received 3 IU oxytocin in 1 L ringer serum, intravenously. Rate of bleeding, need to any surgery interventions, rate of transfusion and changes in hemoglobin and hematocrite were compared between two groups. A total of 400 patients (200 cases in misoprostol group and 200 in oxytocin group) entered to the study. Rate of bleeding > 500 cc was significantly higher in oxytocin group than misoprostol group (33% vs. 19%) (p = 0.005). Also, need to excessive oxytocin for management of postpartum hemorrhage was significantly lower in misoprostol group than oxytocin group (18% vs. 30%) (p = 0.003). Decrease in hematocrite was significantly more observed in oxytocin group than misoprostol group (mean decrease of hematocrite was 1.3 ± 1.6 in misoprostol group and 1.6 ± 2.2 in oxytocin group). Two groups were similar in terms of side-effects. Rectal misoprostol as an uterotonic drug can decrease postpartum hemorrhage and also can prevent from decrease of hemoglobin as compared to oxytocin.

## Introduction

Postpartum hemorrhage is an important cause of maternal morbidity and mortality after delivery. Active management of postpartum hemorrhage by an uterotonic drug decreases the rate of postpartum hemorrhage. Postpartum hemorrhage is usually caused by excessive hemorrhage from placenta implantation area or damage to genital system (1-3). In a survey performed at center of prevention from diseases, hemorrhage was a direct cause of mortality in 18% of maternal deaths. Moreover, hemorrhage is one of the causes of pregnant women hospitalization in NICU. In less-developed countries, hemorrhage in most cases leads to maternal death. Actually, hemorrhage has been known as the most important cause of maternal mortality worldwide and it involves more than half of the cases of postpartum deaths in developing countries (4-6). Bleeding may occur before delivery (such as placenta previa or placental detachment) or more common after delivery (such as bleeding caused from uterine atony or rupture of genital system) (7, 8). Postpartum hemorrhage is defined as bleeding ≥500 cc after completing of labor third phase which is divided into two types of preterm (at first 24 h) and delayed (after first 24 h) (9). One of the common causes of bleeding is uterine inability for suitable contraction after delivery. In the most cases, we can doubt to uterine atony during delivery process. For example, more stretched uterine after delivery is susceptible to being hypotonic. For treatment of postpartum hemorrhage, different drugs with uterotonic characteristics are used (10, 11). First line treatment of mothers with postpartum hemorrhage is administration of oxytocin. However, most studies have shown that administration of oxytocin alone is not enough and it is required to use drug and non-drug methods. If bleeding is not controlled by oxytocic drugs, other methods should be used such as uterine massage with two hands, ligation of intra-iliac artery, uterine artery, uterine compression sutures, angiographic embolization, uterine package, and finally hysterectomy (12, 13).

Prostaglandins are from the drugs used for management of postpartum hemorrhage. Misoprostol (15-deoxy-16-hydroxy-16-methyl prostaglandin E1 [PGE1]) is confirmed for treatment of uterine atony (14). First dose is 250 μg intramuscular and if it is required, the dose is repeated every 15-90 minutes (maximum of 8 doses). Treatment with this combination is not effective as 100% and causes some complications in 20% of women; the complications include hypertension, diarrhea and vomiting, pyrexia, hot flashes, and tachycardia (15). Misoprostol is a synthetic analogue of prostaglandin E1 and an effective uterotonic factor which can be used for treatment of uterine atony. Its advantages in obstetrics and genecology is known and its usage especially for management of postpartum hemorrhage is increasing (16, 17). This combination is extensively used in obstetrics for labor induction, abortion, and cervical ripening. Misoprostol is a synthetic analogue of prostaglandin E1 which its using is very simple; there is no need to intramuscular or intravenous administration and can be kept at room temperature and is easily carried (18). Misoprostol is administered as rectal, vaginal, oral, and sublingual and there is no need to ringer serum or injection. It is saved very easily results and is resistant at room temperature.

Many studies are performed to evaluate the efficacy of rectal misoprostol for prevention of postpartum hemorrhage (19, 20). The study performed in 2004 reported that the use of rectal misoprostol was shown to be an effective first- and second-line treatment for the management of postpartum hemorrhage unresponsive to oxytocin (21). On the other hand, Chong et al. concluded that the drug was not as successful as expected (22). Since different studies have reported contradictory results about the efficacy of rectal misoprostol for prevention of postpartum hemorrhage, and also there are limited studies in this field in our country (Iran), we aimed to perform this study to compare oxytocin with rectal misoprostol for decreasing the postpartum hemorrhage.

## Experimental

This double blind randomized clinical trial study was approved by the Ethics Committee of Zahedan University of Medical Sciences. The study population was all full term pregnant women candidate for vaginal delivery referred to Zahedan Imam Ali Hospital during 2008-2009. The method of sampling was continuous and non-randomized available sampling among the women referred to Zahedan Imam Ali Hospital and based on inclusion and exclusion criteria. They were randomly divided into two groups of rectal misoprostol and oxytocin.

This study was approved by the Ethics Committee of Mashhad University of medical Sciences. The conditions of this study were completely explained for all the patients and if they were consent, a written informed consent was taken from each patient.

Including criteria were primipar or multiparous women with single pregnancy and cephalic presentation who were candidate for vaginal delivery. Exclusion criteria were severe bleeding before delivery, history of previous cesarean, blood pressure ≥ 140/90 mmHg, hemoglobin < 8 g/dL, mal presentation, medical and coagulation disorders (diabetes, epilepsy, heart diseases), history of bronchial asthma, history of complications at previous pregnancy (postpartum hemorrhage, residual placenta), and placenta previa. 

The women in misoprostol group received 400 μg rectal misoprostol after delivery and the women in oxytocin group received 3 IU oxytocin in 1 L ringer serum, intravenously. 

The drugs for treatment of delivery third phase were present at the refrigerator of delivery room including a ringer serum containing 3 mL (30 units) oxytocin and 2 placebo tablets or a ringer serum containing 3 mL normal saline. The drug was administered one minute after placenta delivery and cord clamping. Post-delivery cares were performed such as controlling the blood pressure, pulse, temperature, and rate of bleeding every 15 min during first hour after delivery. 

The rate of hemorrhage at labor third phase was determined by observation estimation considering the amount of blood under the patient. The rate of hemoglobin and hematocrite were measured at hospitalization and also 6 h after delivery and then were recorded. At this interval, the patients were evaluated in terms of possible complications of administered drugs such as vomiting, diarrhea, shivering, pyrexia, and headache). 

Data was analyzed by SPSS software version 13. Quantitative data were shown as mean and standard deviation and qualitative data as frequency. Comparison of qualitative and quantitative data was performed by chi-square and t-student tests. P ≤ 0.05 was considered statistically significant. 

## Results and Discussion

A total of 400 patients (200 cases in misoprostol group and 200 in oxytocin group) entered to the study. Mean age in misoprostol group was 29.7 ± 9.3 yrs and in oxytocin group 28.8 ± 5.6 yrs; no significant difference was observed between two groups (p = 0.278). Also, mean of gravity in misoprostol group was 2.9 ± 1.5 and in oxytocin group 2.7 ± 1.5; there was no significant difference between two groups (p = 0.203). 

**Figure 1:**
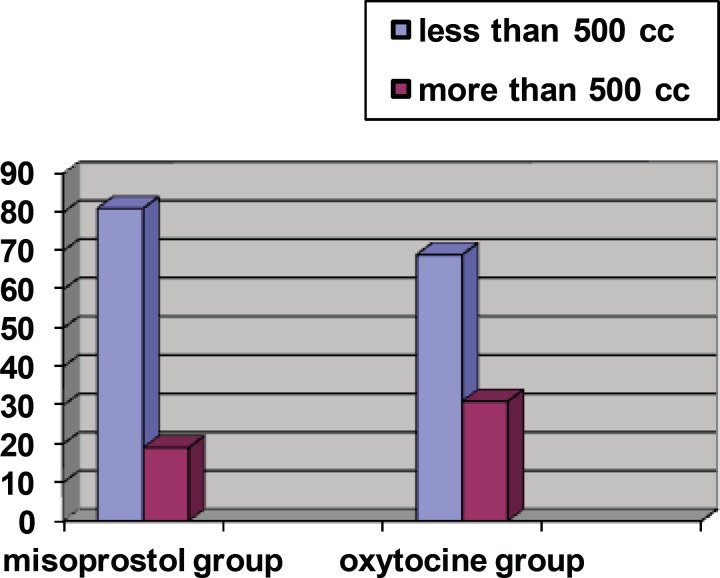
Distribution of hemorrhage frequency in two groups

The rate of bleeding > 500 cc was observed in 31.3% of oxytocin group and 19% of misoprostol group; the difference between two groups was statistically significant (p = 0.005) (Figure 1). 

Also, need to excessive oxytocin for management of postpartum hemorrhage was observed in 30.8% of oxytocin group and 18% of misoprostol group; need to excessive oxytocin was significantly lower in misoprostol group than oxytocin group (p = 0.003). In oxytocin group, 3 patients (1.5%) had need to perform surgery for management of postpartum hemorrhage and in misoprostol group, no patient had need to perform surgery; the difference between two groups was not statistically significant (p = 0.121). In misoprostol group, 8 patients (4%) and in oxytocin group, 14 cases (7.1%) had need to blood injection; although the rate of need to blood injection was higher in oxytocin group than misoprostol group, but the difference was not statistically significant (p = 0.18). 

Table 1 shows the changes in mothers’ hemoglobin and hematocrite before and after delivery in two groups. Before delivery, mean of mothers’ hemoglobin was not statistically different between two groups (p = 0.462). Decrease in hemoglobin was significantly more observed in oxytocin group than misoprostol group (mean decrease of hemoglobin was 1.32 ± 0.9 in oxytocin group and 0.61±0.6 in misoprostol group). Also, mean of mothers’ hematocrite before delivery was not statistically different between two groups (p = 0.06), but decrease in hematocrite was significantly more observed in oxytocin group than misoprostol group (mean decrease of hematocrite was 1.3 ± 1.6 in misoprostol group and 1.6 ± 2.2 in oxytocin group).

**Table 1:** The changes in mothers’ hemoglobin and hematocrite before and after delivery in two groups.

**Groups**	**Misoprostol **		**Oxytocin**		**p-value**
**Variables**					
	**Before**	**After **	**Before**	**After**	
Hemoglobin	11.44 ± 0.7	10.83 ± 1.1	11.38 ± 0.8	10.25 ± 1.1	<0.001
Hematocrite	33.4 ± 1.6	32.1 ± 2.3	33.1 ± 1.8	31.3 ± 2.7	0.005

Table 2 shows the frequency of side-effects in two groups. The most common side-effect was headache, but no significant difference was observed between two groups. No cases of vomiting or diarrhea were reported. Two groups were not statistically different in terms of side-effects.

**Table 2:** The frequency of side-effects in two groups

Groups	**Misoprostol** **N (%) **	**Oxytocin ** **N (%)**	**p-value**
Variables			
Nausea	7 (3.5)	5 (2.5)	0.57
Vomiting	0	0	-
Diarrhea	0	0	-
Shivering	1 (0.5)	0	-
Headache	31 (15.5)	38 (19.2)	0.331
Pyrexia	0	0	-

Misoprostol has been used for more than a decade for prophylaxis and management of postpartum hemorrhage after vaginal birth (23). In this study, we compared the administration of 400 μg of rectal misoprostol with an intravenous infusion of 30 IU of oxytocin as part of the routine active management of postpartum hemorrhage. The outcomes of both groups were comparable and misoprostol was significantly more effective than oxytocin in reducing the incidence of postpartum hemorrhage. Also, need to excessive oxytocin was significantly lower in misoprostol group than oxytocin group.

Several studies are performed about the efficacy of misoprostol for management of postpartum hemorrhage and most of them have shown the effectiveness of misoprostol for management of postpartum hemorrhage (24, 25). This study also showed that misoprostol can decrease postpartum hemorrhage and need to excessive oxytocin.

Chaudhuri *et al. *performed a study in 2010 to compare the efficacy of rectally administered misoprostol with intravenous oxytocin infusion in preventing uterine atony and blood loss during cesarean delivery. A total of 96 and 94 women were analyzed in the misoprostol and oxytocin groups, respectively. Intraoperative and postoperative blood loss was significantly lower in misoprostol group than oxytocin group. The incidence of shivering was significantly higher in misoprostol group (26). In addition, in our study, postpartum hemorrhage was significantly lower in misoprostol group than oxytocin group, but no significant difference was observed between two groups in terms of side-effects.

Nasr *et al. *in 2009 performed a study to compare rectal misoprostol versus intravenous oxytocin for prevention of postpartum hemorrhage. Within 1 min of delivery, the anterior shoulder participants in group 1 received 800 μg of rectal misoprostol and 1 ampoule of normal saline in 5 mL lactated Ringer solution intravenously; group 2 received a rectal placebo tablet and 5 IU of oxytocin in 5 mL lactated Ringer solution intravenously. Two groups were not significantly different in terms of the need for uterotonics, blood transfusion, and 24 h postpartum hemorrhage. Fever was significantly higher among misoprostol patients (18.7% vs 0.8%). There were no significant differences in hemoglobin or hematocrit values 24 h post partum (27). Their results were not in accordance with the findings of our study. This difference may be due to higher dose of misoprostol used in their study.

A randomized controlled trial performed by Mansouri et al. in 2011 evaluated rectal versus oral misoprostol for active management of third stage of labor; they reported that oral misoprostol is associated with significantly more blood loss than rectal; also, shivering and pyrexia was significantly more observed in oral group than rectal group (28). In our study, no significant difference was observed between two groups in terms of side-effects.

Another study evaluating rectally administered misoprostol as a prophylaxis versus conventional intramuscular oxytocin in post partum hemorrhage showed no significant difference between two groups in terms of reducing the incidence of postpartum hemorrhage; therefore reported that rectally administered misoprostol may be effective in the prevention of PPH as an alternative to conventional intramuscular oxytocin (29).

In conclusion, administration of rectal misoprostol appears to be a safe, effective, and cheaper alternative to intravenous oxytocin as a prophylactic uterotonic to decrease the incidence of postpartum hemorrhage. Further studies with larger volume involving high-risk populations are necessary.
